# Benzyl (*E*)-3-(2-bromo-5-meth­oxy­benzyl­idene)dithio­carbazate

**DOI:** 10.1107/S1600536811042826

**Published:** 2011-10-22

**Authors:** Zheng Fan, Yan-Lan Huang, Zhao Wang, Han-Qi Guo, Shang Shan

**Affiliations:** a College of Biological and Environmental Engineering, Zhejiang University of Technology, People’s Republic of China; b College of Chemical Engineering and Materials Science, Zhejiang University of Technology, People’s Republic of China

## Abstract

The title compound, C_16_H_15_BrN_2_OS_2_, was obtained from the condensation reaction of benzyl dithio­carbazate and 2-bromo-5-meth­oxy­lbenzaldehyde. In the mol­ecule, the bromo­meth­oxy­phenyl ring and dithio­carbazate fragment are located on the opposite sides of the C=N double bond, showing the *E* conformation. The dithio­carbazate fragment is approximately planar (r.m.s deviation 0.0187 Å); its mean plane is oriented with respect to the bromo­meth­oxy­phenyl and phenyl rings at 7.60 (12) and 60.08 (9)°, respectively. In the crystal, inversion dimers linked by pairs of N—H⋯S hydrogen bonds occur. A short Br⋯Br contact of 3.5526 (12) Å is observed in the crystal structure.

## Related literature

For the potential application of hydrazone and its derivatives in the biological field, see: Okabe *et al.* (1993 ▶); Hu *et al.* (2001 ▶). For related structures, see: Shan *et al.* (2008*a* ▶,*b* ▶). For the synthesis, see: Hu *et al.* (2001 ▶).
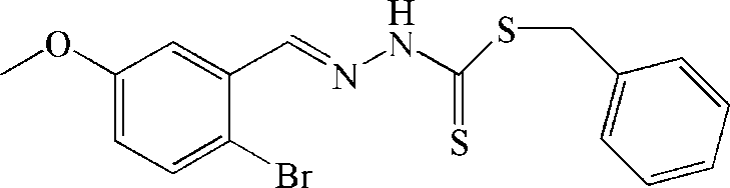


## Experimental

### 

#### Crystal data

C_16_H_15_BrN_2_OS_2_
*M* *_r_* = 395.33
Triclinic, 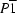

*a* = 6.260 (3) Å
*b* = 11.889 (5) Å
*c* = 12.235 (5) Å
α = 111.931 (5)°
β = 91.725 (4)°
γ = 99.771 (4)°
*V* = 828.1 (6) Å^3^
*Z* = 2
Mo *K*α radiation
μ = 2.74 mm^−1^
*T* = 294 K
0.32 × 0.28 × 0.19 mm

#### Data collection

Rigaku R-AXIS RAPID IP diffractometer
Absorption correction: multi-scan (*ABSCOR*; Higashi, 1995 ▶) *T*_min_ = 0.56, *T*_max_ = 0.72
5637 measured reflections
2988 independent reflections
2379 reflections with *I* > 2σ(*I*)
*R*_int_ = 0.028

#### Refinement

*R*[*F* ^2^ > 2σ(*F* ^2^)] = 0.034
*wR*(*F* ^2^) = 0.069
*S* = 1.02
2988 reflections
201 parameters
H-atom parameters constrained
Δρ_max_ = 0.25 e Å^−3^
Δρ_min_ = −0.30 e Å^−3^

### 

Data collection: *PROCESS-AUTO* (Rigaku, 1998 ▶); cell refinement: *PROCESS-AUTO*; data reduction: *CrystalStructure* (Rigaku/MSC, 2002 ▶); program(s) used to solve structure: *SIR92* (Altomare *et al.*, 1993 ▶); program(s) used to refine structure: *SHELXL97* (Sheldrick, 2008 ▶); molecular graphics: *ORTEP-3 for Windows* (Farrugia, 1997 ▶); software used to prepare material for publication: *WinGX* (Farrugia, 1999 ▶).

## Supplementary Material

Crystal structure: contains datablock(s) I, global. DOI: 10.1107/S1600536811042826/xu5353sup1.cif

Structure factors: contains datablock(s) I. DOI: 10.1107/S1600536811042826/xu5353Isup2.hkl

Supplementary material file. DOI: 10.1107/S1600536811042826/xu5353Isup3.cml

Additional supplementary materials:  crystallographic information; 3D view; checkCIF report

## Figures and Tables

**Table 1:** Hydrogen-bond geometry (Å, °)

*D*—H⋯*A*	*D*—H	H⋯*A*	*D*⋯*A*	*D*—H⋯*A*
N2—H2⋯S1^i^	0.86	2.56	3.402 (3)	167

Symmetry code: (i) 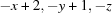.

**Fig. 1.**
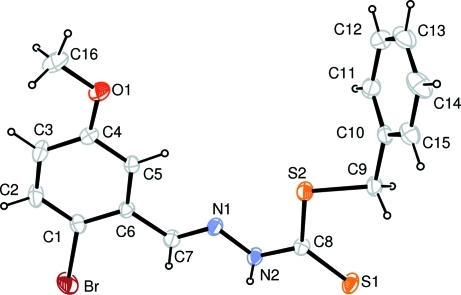
The molecular structure of the title compound with 30% probability displacement (arbitrary spheres for H atoms).

## supplementary crystallographic information

### Comment

Hydrazone and its derivatives have shown the potential application in the biological field (Okabe *et al.*, 1993; Hu *et al.*, 2001). As part of the ongoing investigation on anti-cancer compounds, the title compound has recently been prepared in our laboratory and its crystal structure is presented here.

In the molecules, the methoxylphenyl ring and dithiocarbazate fragment are located on the opposite sides of the C═N double bond, showing the E-configuration. The dithiocarbazate fragment is approximately planar [r.m.s deviation 0.0187 Å]; the mean plane of dithiocarbazate is oriented with respect to the methoxylphenyl and phenyl rings at 7.60 (12) and 60.08 (9)°, similar to those found in related structures (Shan *et al.* 2008*a*, 2008*b*). Intermolecular N—H···S hydrogen bonding is observed in the crystal structure (Table 1). The short Br···Br^i^ contact of 3.5526 (12) Å is also present in the crystal structure [symmetry code: (i) 1-x,-y,-z].

### Experimental

Benzyl dithiocarbazate was synthesized as described previously (Hu *et al.*, 2001). Benzyl dithiocarbazate (0.40 g, 2 mmol) and 2-bromo-5-methoxybenzaldehyde (0.43 g, 2 mmol) were dissolved in ethanol (20 ml), then acetic acid (0.2 ml) was added to the ethanol solution with stirring. The mixture solution was refluxed for 6 h. After cooling to room temperature, microcrystals appeared. The microcrystals were separated from the solution and washed with cold water three times. Recrystallization was performed twice with absolute methanol to obtain colourless single crystals of the title compound.

### Refinement

H atoms were placed in calculated positions with C—H = 0.93–0.97 Å and N—H = 0.86 Å, and refined in riding mode with *U*_iso_(H) = 1.5*U*_eq_(C) for methyl H atoms and 1.2*U*_eq_(C,*N*) for the others.

### Crystal data

**Table:**

C_16_H_15_BrN_2_OS_2_	*Z* = 2
*M**_r_* = 395.33	*F*(000) = 400
Triclinic, *P*1	*D*_x_ = 1.586 Mg m^−^^3^
Hall symbol: -P 1	Mo *K*α radiation, λ = 0.71073 Å
*a* = 6.260 (3) Å	Cell parameters from 2988 reflections
*b* = 11.889 (5) Å	θ = 3.3–25.2°
*c* = 12.235 (5) Å	µ = 2.74 mm^−^^1^
α = 111.931 (5)°	*T* = 294 K
β = 91.725 (4)°	Block, yellow
γ = 99.771 (4)°	0.32 × 0.28 × 0.19 mm
*V* = 828.1 (6) Å^3^	

### Data collection

**Table:**

Rigaku R-AXIS RAPID IP diffractometer	2988 independent reflections
Radiation source: fine-focus sealed tube	2379 reflections with *I* > 2σ(*I*)
graphite	*R*_int_ = 0.028
Detector resolution: 10.0 pixels mm^-1^	θ_max_ = 25.2°, θ_min_ = 3.3°
ω scans	*h* = −5→7
Absorption correction: multi-scan (*ABSCOR*; Higashi, 1995)	*k* = −14→11
*T*_min_ = 0.56, *T*_max_ = 0.72	*l* = −14→14
5637 measured reflections	

### Refinement

**Table:**

Refinement on *F*^2^	Secondary atom site location: difference Fourier map
Least-squares matrix: full	Hydrogen site location: inferred from neighbouring sites
*R*[*F*^2^ > 2σ(*F*^2^)] = 0.034	H-atom parameters constrained
*wR*(*F*^2^) = 0.069	*w* = 1/[σ^2^(*F*_o_^2^) + (0.0276*P*)^2^] where *P* = (*F*_o_^2^ + 2*F*_c_^2^)/3
*S* = 1.02	(Δ/σ)_max_ < 0.001
2988 reflections	Δρ_max_ = 0.25 e Å^−^^3^
201 parameters	Δρ_min_ = −0.30 e Å^−^^3^
0 restraints	Extinction correction: *SHELXL97*, Fc^*^=kFc[1+0.001xFc^2^λ^3^/sin(2θ)]^-1/4^
Primary atom site location: structure-invariant direct methods	Extinction coefficient: 0.0123 (11)

### Special details

**Table:**

Geometry. All e.s.d.'s (except the e.s.d. in the dihedral angle between two l.s. planes) are estimated using the full covariance matrix. The cell e.s.d.'s are taken into account individually in the estimation of e.s.d.'s in distances, angles and torsion angles; correlations between e.s.d.'s in cell parameters are only used when they are defined by crystal symmetry. An approximate (isotropic) treatment of cell e.s.d.'s is used for estimating e.s.d.'s involving l.s. planes.
Refinement. Refinement of *F*^2^ against ALL reflections. The weighted *R*-factor *wR* and goodness of fit *S* are based on *F*^2^, conventional *R*-factors *R* are based on *F*, with *F* set to zero for negative *F*^2^. The threshold expression of *F*^2^ > σ(*F*^2^) is used only for calculating *R*-factors(gt) *etc*. and is not relevant to the choice of reflections for refinement. *R*-factors based on *F*^2^ are statistically about twice as large as those based on *F*, and *R*- factors based on ALL data will be even larger.

### Fractional atomic coordinates and isotropic or equivalent isotropic displacement parameters (Å^2^)

**Table:**

	*x*	*y*	*z*	*U*_iso_*/*U*_eq_	
Br	0.33444 (5)	0.11365 (3)	0.04397 (2)	0.05700 (14)	
S1	1.10460 (10)	0.70610 (6)	0.10224 (6)	0.0458 (2)	
S2	0.72189 (10)	0.76733 (6)	0.24288 (6)	0.0423 (2)	
N1	0.5742 (3)	0.51783 (19)	0.15272 (15)	0.0336 (5)	
N2	0.7654 (3)	0.54749 (19)	0.10904 (16)	0.0356 (5)	
H2	0.8197	0.4905	0.0580	0.043*	
O1	−0.0891 (3)	0.51964 (18)	0.35686 (15)	0.0501 (5)	
C1	0.1988 (4)	0.2431 (2)	0.13964 (19)	0.0366 (6)	
C2	0.0116 (5)	0.2108 (3)	0.1861 (2)	0.0487 (8)	
H2A	−0.0459	0.1278	0.1682	0.058*	
C3	−0.0907 (4)	0.2999 (3)	0.2586 (2)	0.0465 (7)	
H3	−0.2169	0.2778	0.2900	0.056*	
C4	−0.0045 (4)	0.4226 (3)	0.2843 (2)	0.0370 (6)	
C5	0.1811 (4)	0.4548 (2)	0.23569 (19)	0.0341 (6)	
H5	0.2356	0.5378	0.2518	0.041*	
C6	0.2875 (4)	0.3660 (2)	0.16346 (19)	0.0314 (6)	
C7	0.4884 (4)	0.4042 (2)	0.11732 (19)	0.0356 (6)	
H7	0.5525	0.3453	0.0623	0.043*	
C8	0.8663 (4)	0.6654 (2)	0.14642 (18)	0.0319 (6)	
C9	0.8899 (4)	0.9158 (2)	0.2670 (2)	0.0414 (7)	
H9A	1.0306	0.9241	0.3078	0.050*	
H9B	0.9132	0.9233	0.1918	0.050*	
C10	0.7736 (4)	1.0146 (2)	0.3410 (2)	0.0380 (6)	
C11	0.5706 (5)	1.0236 (3)	0.3006 (2)	0.0489 (8)	
H11	0.5048	0.9677	0.2267	0.059*	
C12	0.4647 (5)	1.1145 (3)	0.3690 (3)	0.0587 (8)	
H12	0.3299	1.1210	0.3404	0.070*	
C13	0.5599 (6)	1.1955 (3)	0.4797 (3)	0.0624 (9)	
H13	0.4869	1.2552	0.5269	0.075*	
C14	0.7606 (6)	1.1886 (3)	0.5205 (2)	0.0636 (9)	
H14	0.8250	1.2441	0.5948	0.076*	
C15	0.8682 (5)	1.0989 (3)	0.4509 (2)	0.0487 (7)	
H15	1.0059	1.0953	0.4786	0.058*	
C16	−0.2653 (5)	0.4938 (3)	0.4202 (2)	0.0604 (9)	
H16A	−0.3916	0.4475	0.3655	0.091*	
H16B	−0.2973	0.5700	0.4744	0.091*	
H16C	−0.2260	0.4464	0.4636	0.091*	

### Atomic displacement parameters (Å^2^)

**Table:**

	*U*^11^	*U*^22^	*U*^33^	*U*^12^	*U*^13^	*U*^23^
Br	0.0715 (2)	0.03141 (19)	0.0601 (2)	0.01393 (14)	0.01395 (15)	0.00643 (14)
S1	0.0383 (4)	0.0355 (4)	0.0532 (4)	0.0005 (3)	0.0211 (3)	0.0069 (3)
S2	0.0413 (4)	0.0289 (4)	0.0511 (4)	0.0051 (3)	0.0214 (3)	0.0084 (3)
N1	0.0292 (10)	0.0319 (13)	0.0367 (10)	0.0026 (9)	0.0078 (9)	0.0109 (9)
N2	0.0333 (11)	0.0297 (13)	0.0390 (11)	0.0034 (9)	0.0146 (9)	0.0083 (9)
O1	0.0423 (11)	0.0511 (13)	0.0580 (11)	0.0155 (9)	0.0225 (9)	0.0181 (9)
C1	0.0422 (15)	0.0286 (15)	0.0355 (13)	0.0030 (12)	0.0031 (11)	0.0101 (11)
C2	0.0553 (17)	0.0332 (17)	0.0505 (15)	−0.0061 (14)	0.0095 (14)	0.0140 (13)
C3	0.0389 (15)	0.0474 (19)	0.0507 (15)	−0.0051 (13)	0.0116 (13)	0.0214 (14)
C4	0.0309 (13)	0.0432 (17)	0.0362 (13)	0.0080 (12)	0.0038 (11)	0.0142 (12)
C5	0.0311 (13)	0.0265 (14)	0.0412 (13)	0.0006 (11)	0.0028 (11)	0.0113 (11)
C6	0.0302 (13)	0.0300 (15)	0.0313 (11)	0.0030 (11)	0.0013 (10)	0.0102 (10)
C7	0.0378 (14)	0.0294 (15)	0.0347 (12)	0.0050 (11)	0.0092 (11)	0.0070 (11)
C8	0.0327 (13)	0.0329 (15)	0.0276 (11)	0.0047 (11)	0.0044 (10)	0.0096 (10)
C9	0.0414 (15)	0.0293 (15)	0.0476 (14)	0.0016 (12)	0.0134 (12)	0.0097 (12)
C10	0.0443 (15)	0.0262 (15)	0.0444 (14)	0.0047 (12)	0.0165 (12)	0.0146 (12)
C11	0.0441 (16)	0.0421 (18)	0.0547 (16)	0.0045 (13)	0.0117 (13)	0.0133 (13)
C12	0.0470 (17)	0.054 (2)	0.084 (2)	0.0160 (15)	0.0236 (17)	0.0319 (18)
C13	0.085 (2)	0.043 (2)	0.068 (2)	0.0282 (17)	0.0390 (19)	0.0228 (16)
C14	0.102 (3)	0.0402 (19)	0.0437 (16)	0.0218 (18)	0.0107 (17)	0.0072 (14)
C15	0.0603 (18)	0.0353 (17)	0.0466 (15)	0.0094 (14)	0.0060 (14)	0.0113 (13)
C16	0.0449 (16)	0.085 (3)	0.0553 (16)	0.0222 (16)	0.0221 (14)	0.0258 (16)

### Geometric parameters (Å, °)

**Table:**

Br—C1	1.902 (3)	C6—C7	1.465 (3)
S1—C8	1.658 (3)	C7—H7	0.9300
S2—C8	1.745 (2)	C9—C10	1.505 (4)
S2—C9	1.808 (3)	C9—H9A	0.9700
N1—C7	1.267 (3)	C9—H9B	0.9700
N1—N2	1.370 (3)	C10—C15	1.381 (3)
N2—C8	1.333 (3)	C10—C11	1.385 (4)
N2—H2	0.8600	C11—C12	1.382 (4)
O1—C4	1.368 (3)	C11—H11	0.9300
O1—C16	1.424 (3)	C12—C13	1.378 (4)
C1—C2	1.377 (4)	C12—H12	0.9300
C1—C6	1.387 (4)	C13—C14	1.365 (5)
C2—C3	1.372 (4)	C13—H13	0.9300
C2—H2A	0.9300	C14—C15	1.384 (4)
C3—C4	1.378 (4)	C14—H14	0.9300
C3—H3	0.9300	C15—H15	0.9300
C4—C5	1.382 (3)	C16—H16A	0.9600
C5—C6	1.385 (3)	C16—H16B	0.9600
C5—H5	0.9300	C16—H16C	0.9600
			
C8—S2—C9	101.87 (12)	C10—C9—S2	107.86 (17)
C7—N1—N2	116.98 (19)	C10—C9—H9A	110.1
C8—N2—N1	119.40 (18)	S2—C9—H9A	110.1
C8—N2—H2	120.3	C10—C9—H9B	110.1
N1—N2—H2	120.3	S2—C9—H9B	110.1
C4—O1—C16	118.1 (2)	H9A—C9—H9B	108.4
C2—C1—C6	121.2 (2)	C15—C10—C11	118.5 (2)
C2—C1—Br	117.7 (2)	C15—C10—C9	120.4 (2)
C6—C1—Br	121.16 (18)	C11—C10—C9	121.0 (2)
C3—C2—C1	120.6 (3)	C12—C11—C10	120.8 (3)
C3—C2—H2A	119.7	C12—C11—H11	119.6
C1—C2—H2A	119.7	C10—C11—H11	119.6
C2—C3—C4	119.3 (2)	C13—C12—C11	119.7 (3)
C2—C3—H3	120.3	C13—C12—H12	120.2
C4—C3—H3	120.3	C11—C12—H12	120.2
O1—C4—C3	124.7 (2)	C14—C13—C12	120.3 (3)
O1—C4—C5	115.2 (2)	C14—C13—H13	119.8
C3—C4—C5	120.0 (2)	C12—C13—H13	119.8
C4—C5—C6	121.3 (2)	C13—C14—C15	119.9 (3)
C4—C5—H5	119.3	C13—C14—H14	120.1
C6—C5—H5	119.3	C15—C14—H14	120.1
C5—C6—C1	117.6 (2)	C10—C15—C14	120.8 (3)
C5—C6—C7	119.6 (2)	C10—C15—H15	119.6
C1—C6—C7	122.8 (2)	C14—C15—H15	119.6
N1—C7—C6	119.7 (2)	O1—C16—H16A	109.5
N1—C7—H7	120.1	O1—C16—H16B	109.5
C6—C7—H7	120.1	H16A—C16—H16B	109.5
N2—C8—S1	121.55 (17)	O1—C16—H16C	109.5
N2—C8—S2	113.22 (17)	H16A—C16—H16C	109.5
S1—C8—S2	125.23 (15)	H16B—C16—H16C	109.5

### Hydrogen-bond geometry (Å, °)

**Table:**

*D*—H···*A*	*D*—H	H···*A*	*D*···*A*	*D*—H···*A*
N2—H2···S1^i^	0.86	2.56	3.402 (3)	167

Symmetry codes: (i) −*x*+2, −*y*+1, −*z*.
